# Propylene glycol, a component of electronic cigarette liquid, damages epithelial cells in human small airways

**DOI:** 10.1186/s12931-022-02142-2

**Published:** 2022-08-23

**Authors:** Moegi Komura, Tadashi Sato, Hitomi Yoshikawa, Naoko Arano Nitta, Yohei Suzuki, Kengo Koike, Yuzo Kodama, Kuniaki Seyama, Kazuhisa Takahashi

**Affiliations:** Department of Respiratory Medicine, Juntendo University Graduate School of Medicine, 3-1-3 Hongo, Bunkyo-ku, Tokyo, 113-8431 Japan

**Keywords:** Airway epithelial cell, Apoptosis, Cell cycle, Chronic obstructive pulmonary disease, E-cigarette, E-liquid, Glycerol, Propylene glycol, Small airway

## Abstract

**Background:**

Electronic cigarettes (e-cigarettes) are used worldwide as a substitute for conventional cigarettes. Although they are primarily intended to support smoking cessation, e-cigarettes have been identified as a gateway to smoking habits for young people. Multiple recent reports have described the health effects of inhaling e-cigarettes. E-cigarette liquid (e-liquid) is mainly composed of propylene glycol (PG) and glycerol (Gly), and the aerosol generated by these devices primarily contains these two components. Thus, this study aimed to evaluate the effects of PG and Gly on human small airway epithelial cells (SAECs).

**Methods:**

SAECs were exposed to PG or Gly, and cell proliferation, cell viability, lactate dehydrogenase (LDH) release, DNA damage, cell cycle, and apoptosis were evaluated. Additionally, SAECs derived from chronic obstructive pulmonary disease (COPD) patients (COPD-SAECs) were investigated.

**Results:**

Exposure of SAECs to PG significantly inhibited proliferation (1%, PG, p = 0.021; 2–4% PG, p < 0.0001) and decreased cell viability (1–4% PG, p < 0.0001) in a concentration-dependent manner. Gly elicited similar effects but to a reduced degree as compared to the same concentration of PG. PG also increased LDH release in a concentration-dependent manner (3% PG, p = 0.0055; 4% PG, p < 0.0001), whereas Gly did not show a significant effect on LDH release. SAECs exposed to 4% PG contained more cells that were positive for phosphorylated histone H2AX (p < 0.0001), a marker of DNA damage, and an increased proportion of cells in the G1 phase (p < 0.0001) and increased p21 expression (p = 0.0005). Moreover, caspase 3/7-activated cells and cleaved poly (ADP-ribose) polymerase 1 expression were increased in SAECs exposed to 4% PG (p = 0.0054). Furthermore, comparing COPD-SAECs to SAECs without COPD in PG exposure, cell proliferation, cell viability, DNA damage and apoptosis were significantly greater in COPD-SAECs.

**Conclusion:**

PG damaged SAECs more than Gly. In addition, COPD-SAECs were more susceptible to PG than SAECs without COPD. Usage of e-cigarettes may be harmful to the respiratory system, especially in patients with COPD.

## Background

Electronic cigarettes (e-cigarettes) appeared in the early 2000s in their current form and have since spread worldwide [1]. Toxicant emission from e-cigarette aerosols has been reported to be less than that from conventional cigarette smoke [2, 3]. E-cigarettes are used as devices to support smoking cessation for smokers, including those with chronic obstructive pulmonary disease (COPD) [4–6]. In contrast, these devices have been identified as a potential gateway to smoking habits for young people [7–10]. With the increasing usage of e-cigarettes, the safety of individuals using these devices has become a matter of concern.

In recent years, various hazards of e-cigarette vaping have been reported, and the risks associated with e-cigarette usage have received attention. In 2019, outbreaks of e-cigarette- or vaping-product-associated lung injury (EVALI) were reported in the US [11, 12]. EVALI is currently believed to be caused by tetrahydrocannabinols and vitamin E acetate present in some e-cigarettes [13, 14]. In addition to EVALI, other adverse effects of e-cigarette inhalation have been reported. For example, e-liquid exposure causes COPD features in the lungs of mice [15], disruption of lung lipid homeostasis and impaired innate immunity in mice [16], and disruption of the protease-antiprotease balance [17]. Thus, e-cigarettes are by no means safer than conventional cigarettes.

In e-cigarette devices, e-cigarette liquids (e-liquids) are mainly composed of propylene glycol (PG) and glycerol (Gly), which add flavours and nicotine, and are heated in the device to generate aerosols without combustion [1]. PG and Gly are used as food additives and drug solvents and have been tested for safety; however, the safety of inhalation of these components has not been confirmed. PG and Gly are known to be oxidised to aldehydes on heating, which have been subsequently detected in e-cigarette aerosols [18]. Although some studies have evaluated the toxic effects of PG and Gly on human bronchial epithelial cells [19–21], most of these studies were designed to assess the exposure to e-cigarette vapour or PG/Gly mixtures; hence, the pure effects of PG and Gly without aldehydes remain unclear.

Thus, in the current study, we investigated whether PG and Gly themselves affect small airway epithelial cells (SAEC) proliferation and viability. Moreover, to investigate these reactions in cells that had already been damaged by cigarette smoke, we assayed the effects in SAECs derived from COPD patients (COPD-SAECs).

## Methods

### Cell culture and treatment

Commercially available primary human SAECs and COPD-SAECs were used in this study. SAECs and COPD-SAECs were purchased from LONZA (Basel, Switzerland). For SAECs, cells from healthy and non-smoker donors were selected. Both sets of cells were cultured in small airway epithelial cell growth medium (LONZA) in accordance with the manufacturer’s instructions. Cells from passages 2–4 were used for the assays. Three batches of SAECs and COPD-SAECs were evaluated on two separate occasions in each experiment. PG (FUJIFILM WAKO Pure Chemical Corporation, Osaka, Japan) or Gly (FUJIFILM WAKO Pure Chemical Corporation) were added into the culture medium at concentrations of 0–4%.

### Cell proliferation and viability assays

Cells were cultured in 96-well plates (5000 cells/well) with 100 μL of culture medium in each well. After 24 h of seeding, the medium was replaced with medium containing PG or Gly. Cells were assessed in triplicate in each condition. Each well was divided into four sections, and pictures of each section were taken every 3 h up to 96 h by using IncuCyte Zoom (Essen Bioscience, Ann Arbor, MI, USA) at 10 × magnification. Cell surface areas were calculated using the IncuCyte Zoom software (Essen Bioscience).

Cells were cultured in 96-well plates (5000 cells/well) with 100 μL of culture medium in each well. After 24 h of seeding, the medium was replaced with medium containing PG or Gly. Cells were assessed in triplicate in each condition. Cell viability was measured after 96 h exposure to 0–4% PG or Gly using the Cell Counting Kit-8 (CCK-8; Dojindo Laboratories, Kumamoto, Japan) in accordance with the manufacturer’s instructions. Lactate dehydrogenase (LDH) release from the cells was measured after 24 h exposure to 0–4% PG or Gly using the Cytotoxicity LDH Assay Kit-WST (Dojindo Laboratories) in accordance with the manufacturer’s instructions.

### DNA damage assay

Cells were cultured in 2-well chamber slides (50,000 cells/well). After reaching approximately 70% confluence, the cells were treated with PG or Gly for 24 h and then fixed with 4% paraformaldehyde containing 0.1% Triton-X and 250 mM HEPES for 15 min. For permeabilization, 1% Triton-X was used. The blocking solution, which contained the antibody of phosphorylated histone H2AX (γH2AX) and the secondary antibody, was included in the DNA damage detection kit (Dojindo Laboratories) and was used according to the manufacturer’s instructions. Images were captured with a fluorescence microscope TCS-SP5 (Leica Microsystems, Wetzlar, Germany). All images were taken at 40 × magnification.

### Cell cycle assay

Cells were cultured in 6-cm dishes (200,000 cells/dish) to approximately 70% confluence and then treated with PG or Gly for 24 h. After treatment, cells were harvested using 0.025% trypsin and centrifuged for trypsin removal. Subsequently, cells were treated with Cell Cycle Assay Solution Deep Red (Dojindo Laboratories) and incubated for 15 min at 37 °C according to the manufacturer’s instructions. Data were obtained using FACSCelesta (Becton Dickinson, Franklin Lakes, NJ, USA) counting up to 10,000 cells and analysed using FlowJo software (Becton Dickinson).

### Apoptosis assay

Cells were cultured in 96-well plates (5000 cells/well) and 0.1% IncuCyte Caspase-3/7 Green Reagent (Essen Bioscience) was added to the medium with PG or Gly to detect caspase 3/7-activated cells. Cells were assessed in triplicate in each condition. Immunofluorescent images were taken at 3 h intervals up to 24 h at 10 × magnification using IncuCyte Zoom (Essen Bioscience). Caspase 3/7-positive cells were counted using the IncuCyte Zoom software (Essen Bioscience).

### Western blot

Cells were cultured in 6-cm dishes (100,000 cells/dish). Approximately 70% confluent cells were exposed to 4% PG or Gly for 24 h, and proteins were extracted using RIPA buffer containing protease and phosphatase inhibitors. Protein content was measured using the BCA Protein Assay Kit (Thermo Fisher Scientific, Waltham, MA, USA). Twenty micrograms of total protein was fractionated by electrophoresis on 4–20% Mini-PROTEAN TGX Gels (Bio-Rad Laboratories, Hercules, CA, USA). Trans-Blot Turbo Transfer Pack (Bio-Rad Laboratories) was used for transferring proteins, and membranes were blocked with PVDF Blocking Reagent for Can Get Signal (TOYOBO, Osaka, Japan) for 1 h between 25 °C and 28 °C. Membranes were then incubated with anti-p21 monoclonal antibody (1:200; Cat. No. sc6246, Santa Cruz Biotechnology, Dallas, TX, USA), anti-cleaved poly (ADP-ribose) polymerase 1 (PARP1, 1:1000; Cat. No. ab32064, Abcam, Cambridge, MA, USA), or anti-β actin (1:1000; Cat. No. 4970, Cell Signalling Technology, Danvers, MA, USA) at 4 °C overnight, followed by incubation with the appropriate horseradish peroxidase-conjugated secondary antibodies (1:5000; GE Healthcare, Pittsburgh, PA, USA) for 1 h at room temperature. Clarity western ECL substrate (Bio-Rad Laboratories) was used to detect the signals, which were captured using a ChemiDoc Touch (Bio-Rad Laboratories) and quantified using ImageLab software (Bio-Rad Laboratories).

### Statistical analysis

Data were analysed using GraphPad Prism 8 (GraphPad Software, San Diego, CA, USA). Two-way ANOVA or unpaired-t test was used for analyses. Data are shown as mean ± standard error of the mean (SEM). Differences were considered significant at *p* < 0.05.

## Results

### PG inhibits cell proliferation and cell viability in SAECs

**Fig. 1:**
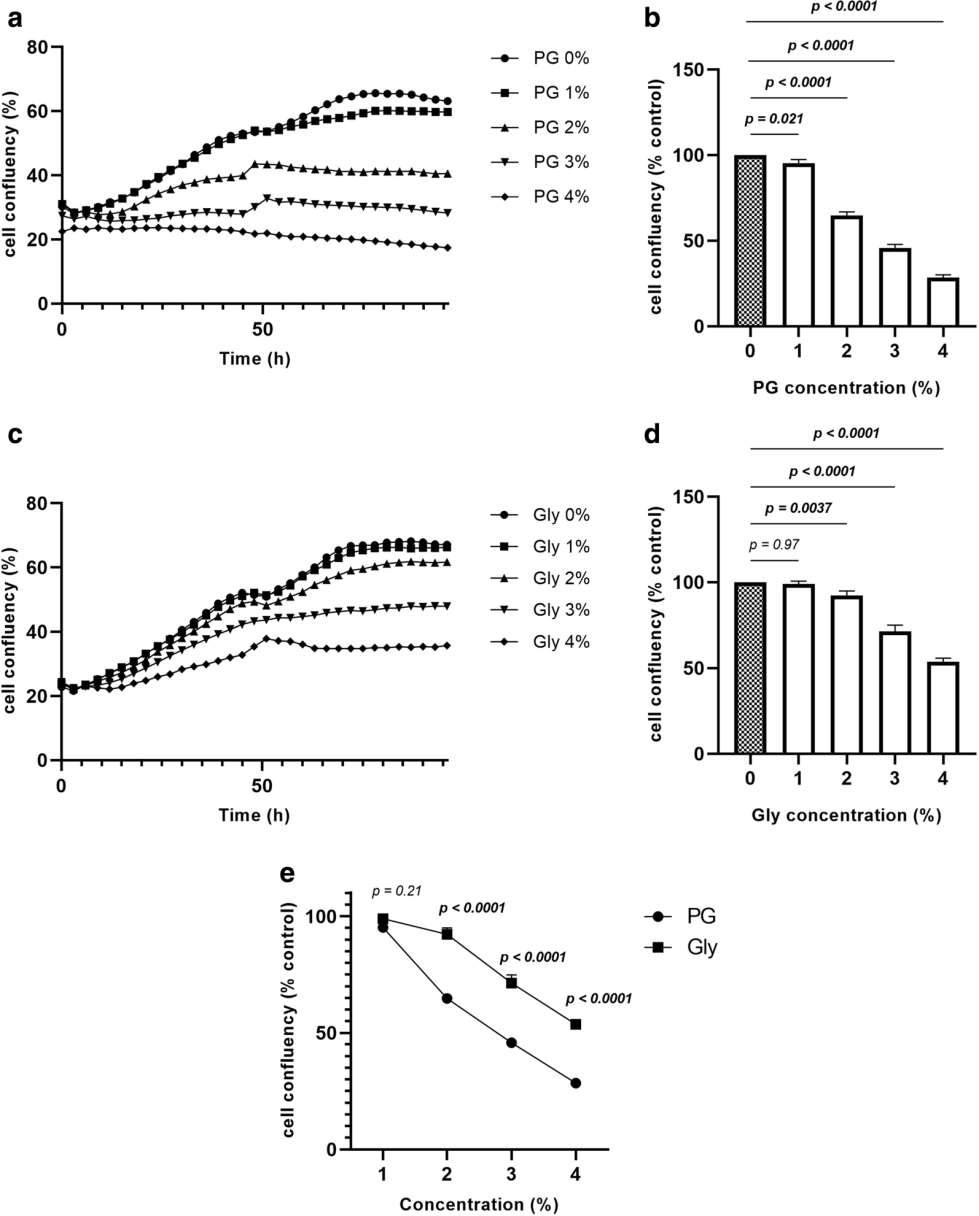
PG inhibits cell proliferation in a concentration-dependent manner. SAECs were cultured with 0–4% PG or Gly. Cell confluency was assessed every 3 h up to 96 h using IncuCyte Zoom. **A** Effect of PG on cell confluency, time-course. **B** Effect of PG on cell confluency at 96 h. Data were compared with control (indicated as a grey bar). **C** Effect of Gly on cell proliferation, time-course. **D** Effect of Gly on cell confluency at 96 h. Data were compared with control (indicated as a grey bar). **E** Comparison of cell confluency after PG exposure and Gly exposure. Three batches of SAECs were evaluated on two separate occasions. Values are mean ± SEM. Statistical significance was determined using two-way ANOVA. *PG* propylene glycol, *Gly* glycerol, *SAECs* small airway epithelial cells, *SEM* standard error of the mean

To assess the effects of PG and Gly on cell proliferation, we first exposed SAECs to 0–4% PG or Gly and measured the change in cell surface area by using IncuCyte. We found that PG exposure inhibited cell proliferation in a concentration-dependent manner, with a decrease in cell surface area after exposure to 1–4% PG (Fig. 1A and B). In contrast, exposure to 2–4% Gly inhibited cell proliferation (Fig. 1C and D). Comparing the effect of PG and Gly exposure on cell proliferation, 2–4% PG affected SAECs more significantly than Gly at the same concentration of (Fig. 1E).

**Fig. 2:**
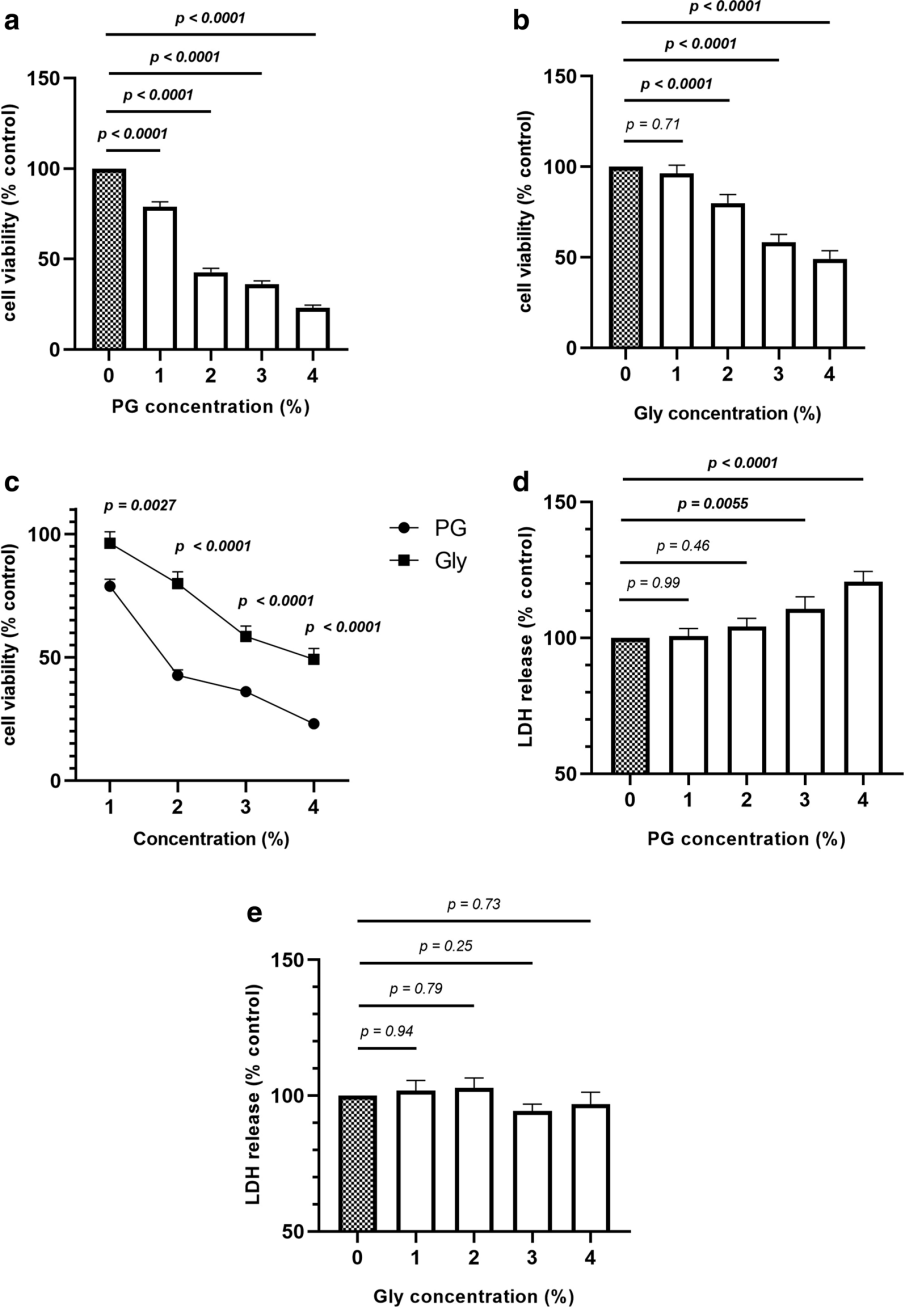
PG decreases cell viability in a concentration-dependent manner. Cell viability was evaluated by using CCK-8 and LDH assays. The CCK-8 assay was conducted after 96 h exposure to 0–4% PG or Gly. The LDH assay was conducted after 24 h exposure to 0–4% PG or Gly. **A** Effect of PG on cell viability by CCK-8. **B** Effect of Gly on cell viability by CCK-8. **C** Comparison of cell viability after PG exposure and Gly exposure. **D** Effect of PG on LDH release. **E** Effect of Gly on LDH release. Data are expressed as a relative value to control (indicated as a grey bar). Three batches of SAECs were evaluated on two separate occasions. Values are mean ± SEM. Statistical significance was determined using two-way ANOVA. *PG* propylene glycol, *Gly* glycerol, *CCK-8* cell counting kit-8, *LDH* lactate dehydrogenase, *SAECs* small airway epithelial cells, *SEM* standard error of the mean

We then evaluated cell viability by using CCK-8 and LDH assays. Cell viability as measured by CCK-8 decreased significantly after PG exposure in a concentration-dependent manner (Fig. 2A). 2–4% Gly also decreased cell viability (Fig. 2B), but to a lesser extent than the same concentration of PG (Fig. 2C). LDH levels in the cell culture supernatant increased significantly with 3–4% PG exposure, but not with Gly exposure, even at the same concentration (Fig. 2D and E).

### PG causes DNA damage and induces cell cycle arrest in SAECs

**Fig. 3:**
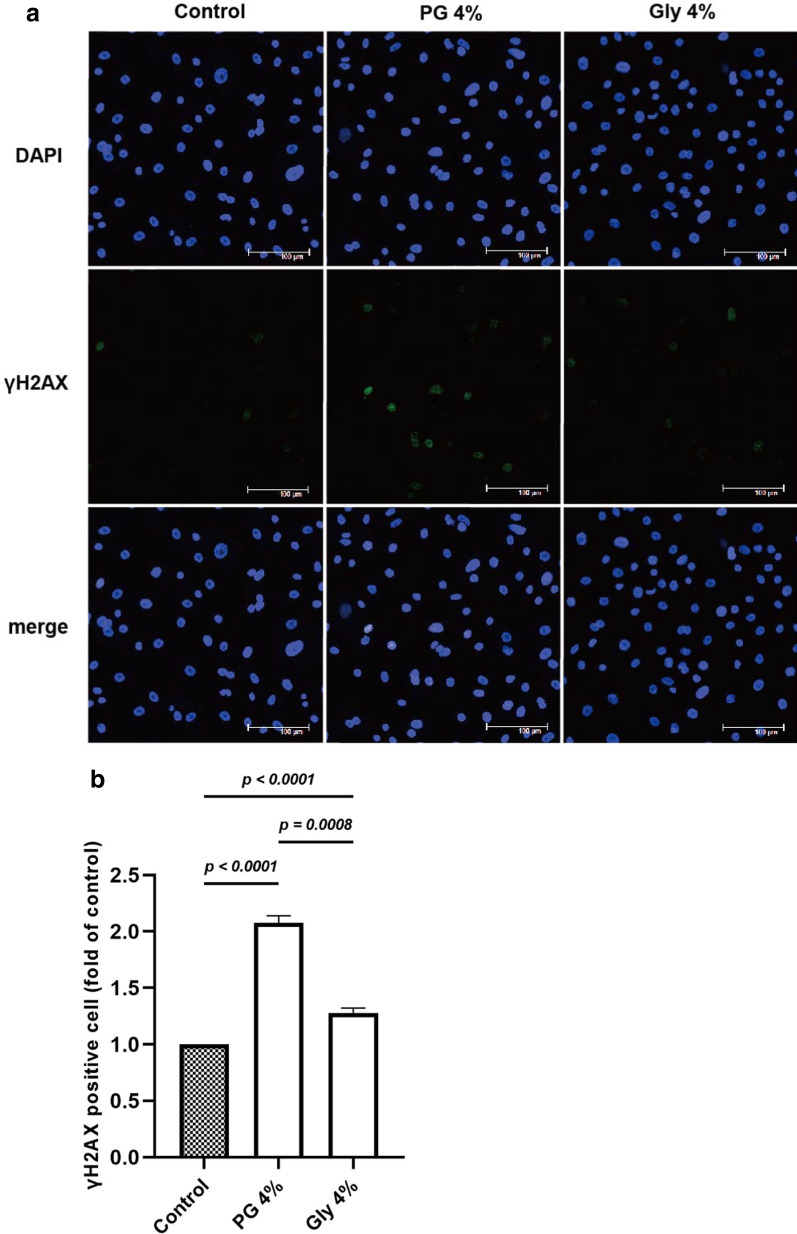
PG induces DNA damage. SAECs were cultured with 4% PG or Gly for 24 h. **A** Representative immunofluorescent images of the antibody reaction against γH2AX. Green signals indicate γH2AX-positive cells. Scale bar = 100 µm. **B** Ratio of γH2AX-positive cells to control (without PG and Gly). Three batches of SAECs were evaluated on two separate occasions. Values are mean ± SEM. Statistical significance was determined using two-way ANOVA. *PG* propylene glycol, *Gly* glycerol, *SAECs* small airway epithelial cells, *SEM* standard error of the mean

**Fig. 4:**
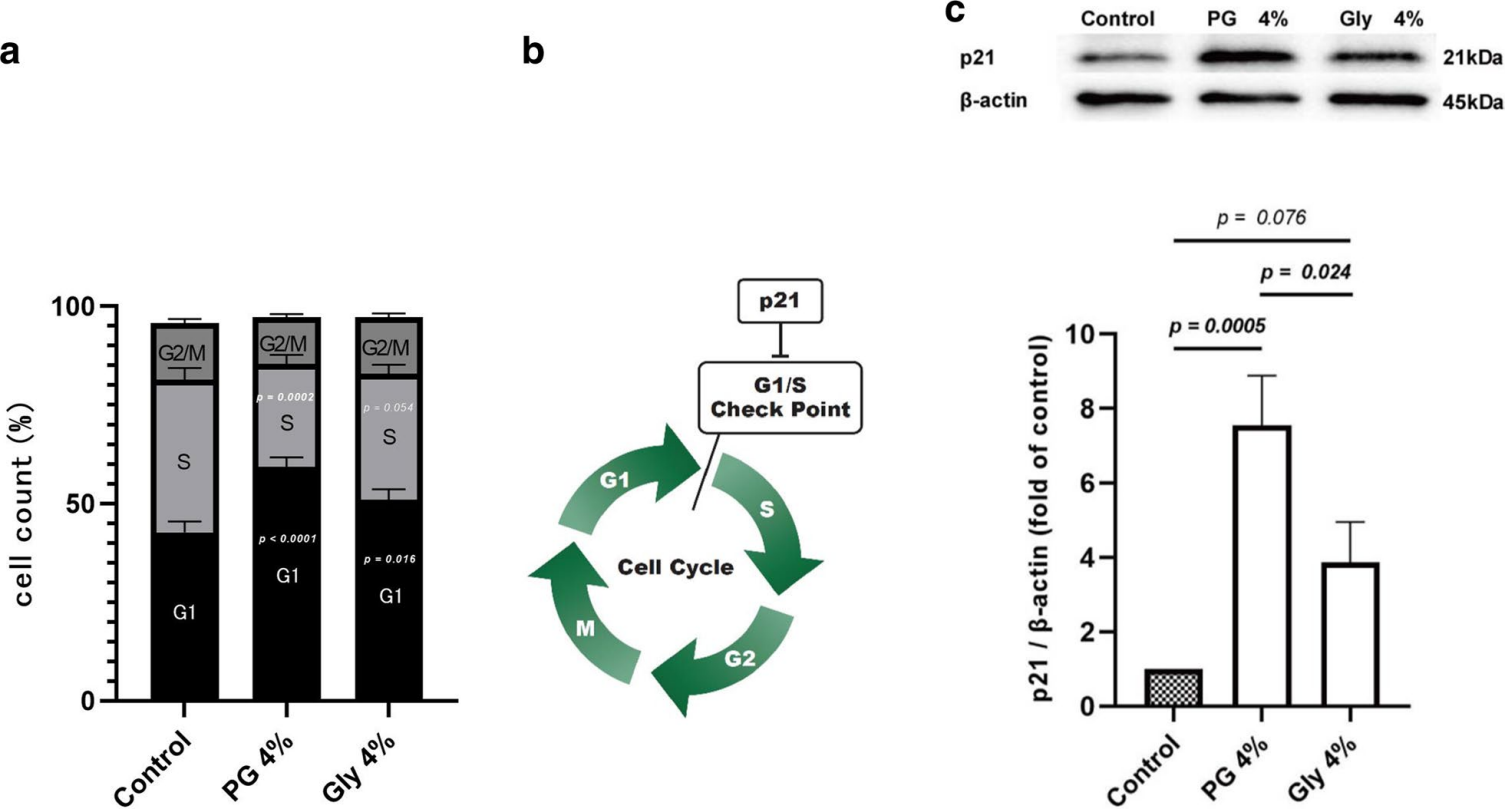
PG induces cell cycle arrest at the G1 phase. SAECs were cultured with 4% PG or Gly for 24 h. **A** Distribution of cell cycle phase in each group by flow cytometry. Data were compared with the control sample in the same phase respectively. **B** Relationship of p21 and cell cycle. **C** Effect of PG and Gly on p21 expression. Representative western blot images are shown. β-actin was used as a loading control. Band size is indicated on the upper right. Densitometric analysis of p21 expression, normalized to that of β-actin. Data are expressed as a relative value to control (without PG and Gly, indicated as a grey bar). Three batches of SAECs were evaluated on two separate occasions. Values are mean ± SEM. Statistical significance was determined using two-way ANOVA. *PG* propylene glycol, *Gly* glycerol, *SAECs* small airway epithelial cells, *SEM* standard error of the mean

To investigate the mechanism(s) underlying the inhibition of cell proliferation and viability, we evaluated DNA damage and the cell cycle. After 24 h of exposure of 4% PG or Gly, γH2AX was more highly expressed in PG-exposed cells than in untreated cells (Fig. 3A and B). Additionally, Gly-treated cells showed an increase in the number of γH2AX-positive cells; however, the number was markedly reduced compared to cells treated with PG (Fig. 3B). We then performed cell cycle analysis by measuring DNA content distribution after 24 h exposure of 4% PG or Gly. PG-exposed cells included more cells in the G1 phase and fewer cells in the S phase; these findings were more prominent in PG-treated cells than in Gly-treated cells (Fig. 4A). Moreover, after 24 h of exposure to 4% PG or Gly, the protein levels of p21, a cyclin-dependent kinase inhibitor that regulates the cell cycle in the G1 phase [22] (Fig. 4B), was significantly increased by PG exposure compared with control, but not by Gly exposure (Fig. 4C). Furthermore, PG-treated cells showed higher protein levels of p21 compared with Gly-treated cells (Fig. 4C).

### PG induces apoptosis in SAECs

**Fig. 5:**
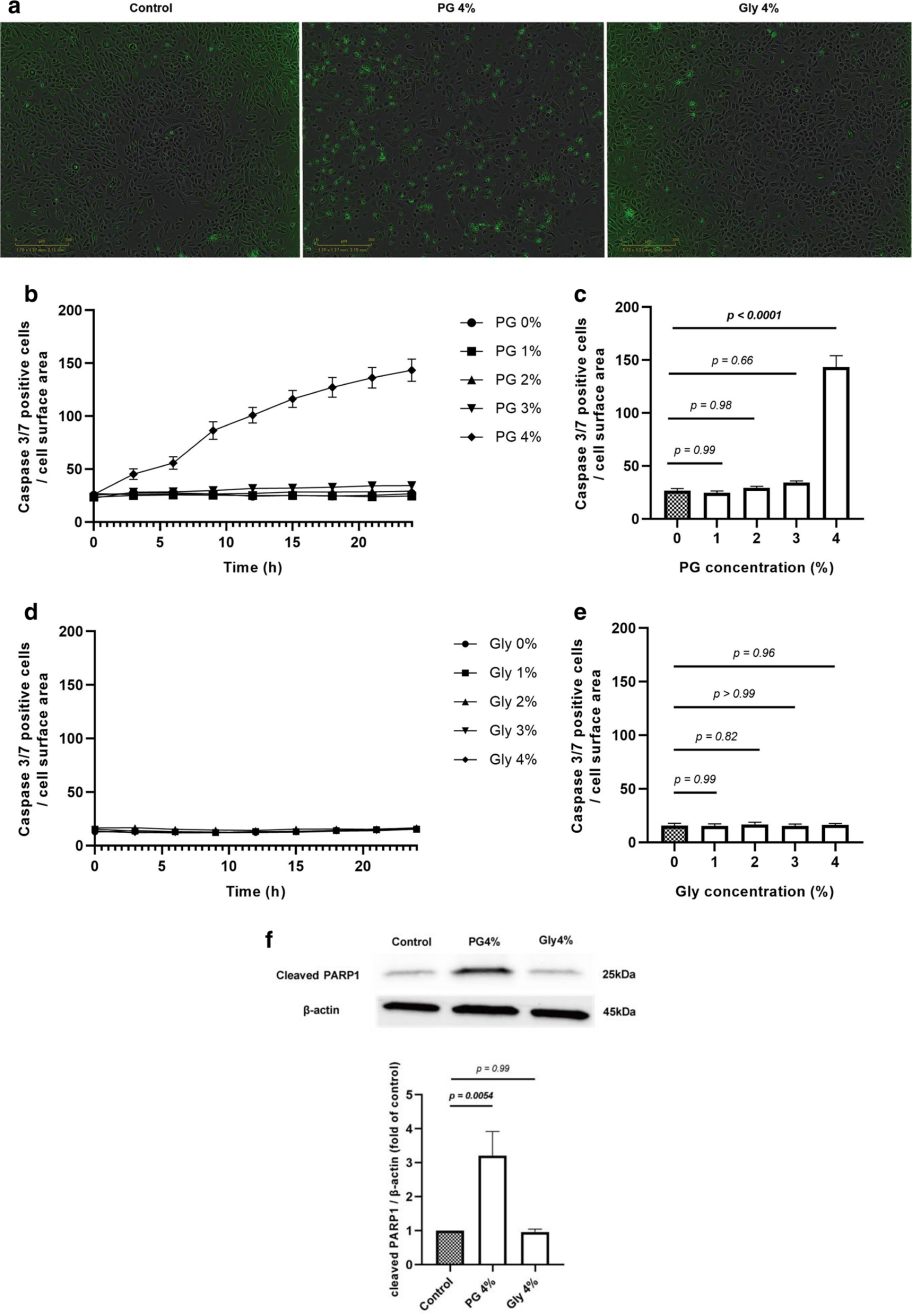
PG induces cell apoptosis. SAECs were cultured with 0–4% PG or Gly. Caspase 3/7-positive cells were assessed every 3 h up to 24 h using IncuCyte Zoom. Data were adjusted with cell surface area. **A** Representative images of SAECs exposure to 4% PG or Gly at 24 h. Green signals indicate caspase 3/7-positive cells. Scale bar = 300 µm. **B** Effect of PG on caspase 3/7-positive cell count, time-course. **C** Effect of PG on caspase 3/7-positive cell count at 24 h. Data are expressed as a relative value to control (indicated as a grey bar). **D** Effect of Gly on caspase 3/7-positive cell count, time-course. **E** Effect of Gly on caspase 3/7-positive cell count at 24 h. Data are expressed as a relative value to control (indicated as a grey bar). **F** Effect of PG and Gly on cleaved PARP1 expression at 24 h. Representative western blot images are shown. β-actin was used as a loading control. Band size is indicated on the upper right. Densitometric analysis of cleaved PARP1 expression, normalized to that of β-actin. Data are expressed as a relative value to control (without PG and Gly, indicated as a grey bar). Three batches of SAECs were evaluated on two separate occasions. Values are mean ± SEM. Statistical significance was determined using Two-way ANOVA. *PG* propylene glycol, *Gly* glycerol, *SAECs* small airway epithelial cells, *PARP* poly (ADP-ribose) polymerase, *SEM* standard error of the mean

Since PG induced cell cycle arrest in the G1 phase, we next examined whether PG leads to apoptosis by counting caspase 3/7-activated cells at different time points. After PG or Gly exposure at concentrations of 0–4% for 24 h, we found that 4% PG significantly induced cell apoptosis in a time-dependent manner (Fig. 5A–C), whereas Gly did not (Fig. 5A, D, and E). Then, we evaluated the expression of cleaved PARP1 protein. After 24 h of exposure to 4% PG, the cleaved PARP1 level was significantly elevated; however, Gly treatment did not cause elevation of the protein level (Fig. 5F). These results indicate that a relatively high concentration of PG induced apoptosis whereas Gly did not have this effect even at the same concentration.

### COPD-SAECs are more susceptible to PG than SAECs without COPD

**Fig. 6:**
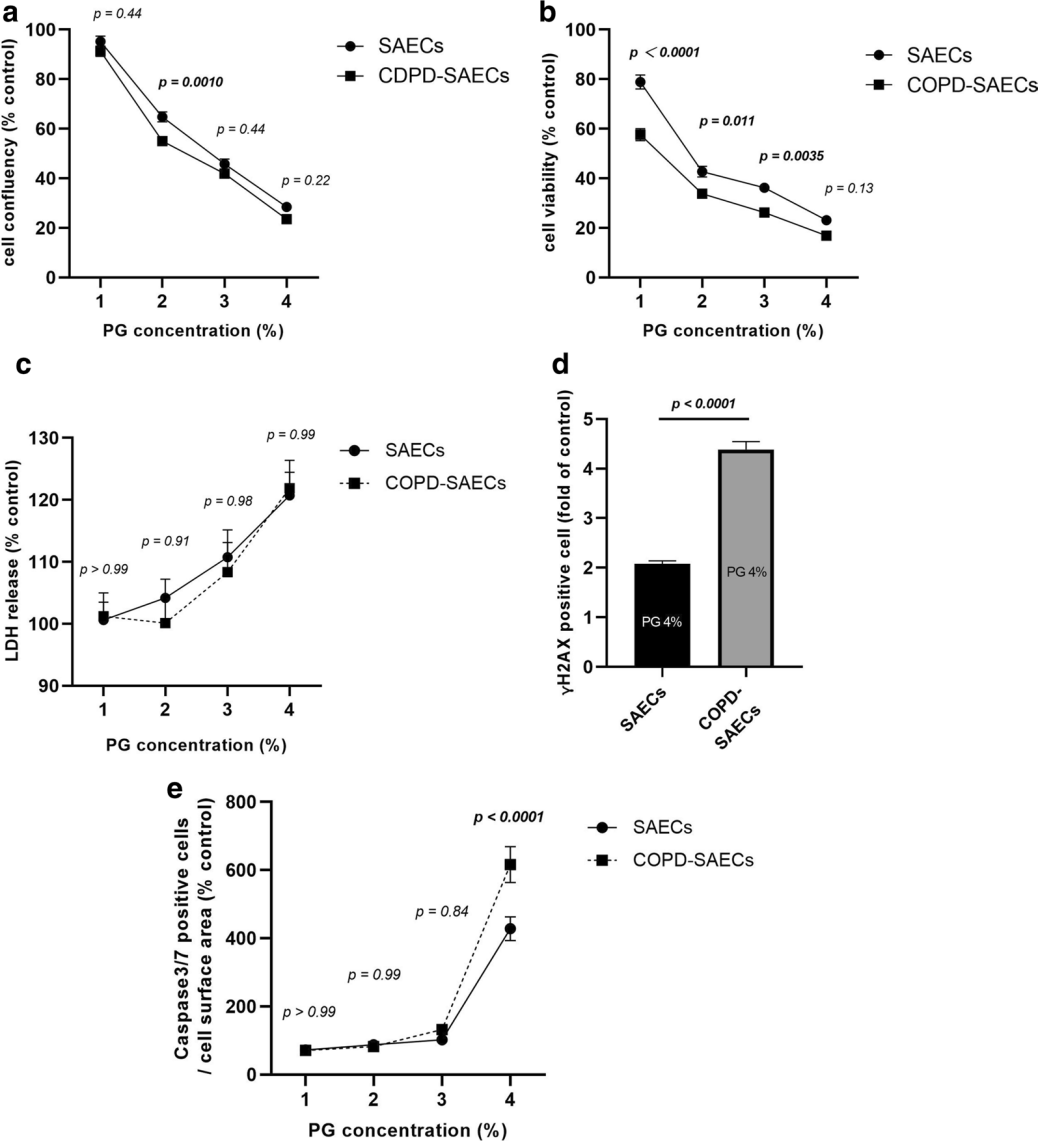
COPD-SAECs are affected more by PG exposure. **A** Effect of PG on cell confluency at 96 h. **B** Effect of PG on cell viability by CCK-8. **C** Effect of PG on LDH release. **D** Effect of 4% PG on the ratio of γH2AX-positive cells at 24 h. **E** Effect of PG on caspase 3/7-positive cells at 24 h. Three batches of SAECs and COPD-SAECs were evaluated on two separate occasions. Values are mean ± SEM. Data are adjusted with control of each cell (without PG exposure). Statistical significance was determined using Two-way ANOVA and unpaired t- test. *PG* propylene glycol, *SAECs* small airway epithelial cells, *COPD* chronic obstructive pulmonary disease, *CCK-8* cell counting kit-8, *LDH* lactate dehydrogenase, *SEM* standard error of the mean

In the current series of experiments, we found that PG has prominent effects on SAECs. We then compared SAECs from healthy donors and COPD-SAECs to evaluate the differences in their reactions to PG exposure. Regarding cell proliferation evaluated by IncuCyte, the cell surface area of COPD-SAECs was significantly decreased following exposure with 2% PG (Fig. 6A). Furthermore, COPD-SAECs showed significant inhibition of cell viability based on CCK-8 with 1%-3% PG (Fig. 6B). LDH levels were almost the same in both sets of cells (Fig. 6C). The ratio of γH2AX-positive and caspase 3/7-positive cells was significantly higher in COPD-SAECs compared to that in control cells after exposure to 4% PG (Fig. 6D and E).

## Discussion

Among the e-liquid components, PG inhibited cell proliferation and decreased cell viability in SAECs, whereas the effects of Gly were less severe than those of PG. PG exposure further increased the proportion of γH2AX-positive cells, which indicate DNA damage, and led to cell cycle arrest at the G1 phase; in contrast, Gly had a relatively smaller effect. The effects of PG exposure eventually induced apoptosis, which was confirmed by the increased number of caspase 3/7-positive cells and cleaved PARP1 expression in SAECs.

E-liquids are mainly composed of PG and Gly, along with added flavours and nicotine. PG is related to the taste, and Gly is added to facilitate aerosol generation. Thus, the main vapour contains large amounts of PG and Gly. The composition ratio of PG and Gly can be adjusted according to personal preference [23–25]. However, there is no definitive information on the long-term health effects of these compounds, especially in the respiratory system.

Evidence regarding the biological effects of e-cigarette use has been increasing recently. There are several reports that PG and Gly inhibit cell proliferation and viability in various types of cells [20, 26, 27]. However, most of these studies used a vapour or PG/Gly mixture. PG and Gly undergo heating to form aerosols, but the oxidation reaction during this process results in the generation of aldehydes [18], which are harmful substances [28]. To reduce the generation of these compounds, products that aerosolise PG and Gly at low temperatures have been introduced. Therefore, we believe that the direct effects of PG and Gly, especially in respiratory cells, should be evaluated. We had previously reported that SAECs are more susceptible to cigarette smoke extract than large airway epithelial cells [29]. However, none of the previous studies evaluated the effects of PG and Gly by using SAECs; thus, the present study is the first to show the direct effects of PG and Gly on SAECs. The findings of this study clearly showed an inhibition of cell proliferation, decrease in the cell viability and increase in LDH release following PG exposure. Gly exposure also affected cell proliferation and viability, however the effects were clearly less severe than that of PG. Moreover, an increase in LDH release was not observed with Gly. Therefore, PG, the main component of e-liquid, is harmful to the human respiratory system.

Then, we evaluated DNA damage and cell cycle arrest. When DNA damage causes double-strand breaks, phosphorylation of Ser139 of H2AX, a histone H2A variant, occurs and leads to the formation of γH2AX [30]. DNA damaged cells are monitored at the G1/S checkpoint of the cell cycle, which leads to cell cycle arrest or cell apoptosis [31]. The transition of the G1 phase to the S phase in the cell cycle involves cyclin/CDK complexes, which are inhibited by p21 [22]. The current results showed that PG exposure increased the number of γH2AX-positive cells and G1 phase cells and decreased the number of cells in S phase, with more increased expression of p21 in PG-exposed SAECs. These results indicate that PG induced DNA damage and cell cycle arrest at the G1 phase, which led to the inhibition of cell proliferation. Additionally, exposure to Gly increased the number of γH2AX-positive cells and the number of cells in the G1 phase; however, its effect was much less than that of PG. This result for Gly is consistent with the results of the cell proliferation and viability assays.

Cellular DNA damage can trigger apoptosis in addition to cell cycle arrest [31, 32]. Damage to the cellular DNA promotes apoptosis by caspase 3/7 activation and PARP cleavage [33]. E-liquid or e-cigarette vapour condensate has been reported to cause apoptosis in human alveolar macrophages [34]; however, no previous report has described the relationship between PG/Gly and apoptosis in airway cells. The current study showed that PG exposure increased caspase 3/7-positive signals and cleaved PARP1 expression, indicating that PG clearly induced apoptosis in SAECs. Contrastingly, Gly did not induce apoptosis at all, suggesting that the effects of Gly are insufficient to induce apoptosis compared with the same concentration of PG.

As mentioned earlier, e-cigarettes are often used as a supportive device for smoking cessation, and COPD patients have higher odds of using e-cigarettes compared to non-COPD patients [6]. Defective regenerative ability in the airway and epithelial barrier dysfunction has been previously reported in COPD patients [35–37]. In the current study, COPD-SAECs revealed inhibition of cell confluence and a significant decrease of cell viability compared with SAECs derived from healthy donors. In contrast, LDH levels were not different, which may indicate the same degree of cellular membrane injury. However, cellular DNA damage and apoptosis were observed more clearly at relatively high concentrations of PG exposure in COPD-SAECs. These results indicate that COPD-SAECs have a higher susceptibility to PG than control SAECs. Therefore, we believe that e-cigarettes should not be recommended, especially for COPD patients, even when quitting conventional cigarettes.

A limitation of this study was that the actual concentrations of PG and Gly in the airway were uncertain. In this study, the concentrations of PG and Gly ranged from 0.5 to 4%. The predicted deposition of the e-cigarette mainstream aerosol is 15–45% [38]. When 1 mL of e-liquid composed of PG and Gly was inhaled, assuming that the total liquid on the airway surface was 3 mL, the e-liquid concentration in the airway surface liquid was estimated to be 5–15% [20]. If the e-liquid contained 50% PG and Gly each, both components would be in the range of 2.5–7.5%, which is not substantially different from the concentrations used in this study. Finally, the COPD-SAECs used in the current study were commercially available, and we did not have clinical information regarding their donors. Thus, we could not investigate the relationship between the experimental results and clinical information, such as smoking history and COPD severity.

## Conclusion

Among the e-liquid components, PG predominantly inhibited cell proliferation and viability by enhancing DNA damage and cell cycle arrest in SAECs. PG also promoted apoptosis in SAECs, whereas Gly did not. This study presents the first evidence demonstrating that PG can injure SAECs directly and is a harmful agent in the human respiratory system. In addition, some of the results were more prominent in cells from COPD patients, suggesting that e-cigarette inhalation may be more harmful to COPD patients.

## Data Availability

The datasets used and analysed during the current study are available from the corresponding author on reasonable request.

## Abbreviations

COPD: Chronic obstructive pulmonary disease
EVALI: E-cigarette- or vaping-product-associated lung injury
Gly: Glycerol
HBEC: Human bronchial epithelial cells
LDH: Lactate dehydrogenase
γH2AX: Phosphorylated histone H2AX
PARP: Poly (ADP-ribose) polymerase
PG: Propylene glycol
SAEC: Small airway epithelial cells
SEM: Standard error of the mean
